# Hydrogel-Gated FETs in Neuromorphic Computing to Mimic Biological Signal: A Review

**DOI:** 10.3390/bios14030150

**Published:** 2024-03-19

**Authors:** Sankar Prasad Bag, Suyoung Lee, Jaeyoon Song, Jinsink Kim

**Affiliations:** Department of Biomedical Engineering, College of Life Science and Biotechnology, Dongguk University, Seoul 04620, Republic of Korea; sankar2022@dongguk.edu (S.P.B.); suyoung7541@dongguk.edu (S.L.); thdwodbs95@dongguk.edu (J.S.)

**Keywords:** synaptic transistor, organic electrochemical FET, hydrogel, biodegradable, biocompatibility, neurodegenerative disorder

## Abstract

Hydrogel-gated synaptic transistors offer unique advantages, including biocompatibility, tunable electrical properties, being biodegradable, and having an ability to mimic biological synaptic plasticity. For processing massive data with ultralow power consumption due to high parallelism and human brain-like processing abilities, synaptic transistors have been widely considered for replacing von Neumann architecture-based traditional computers due to the parting of memory and control units. The crucial components mimic the complex biological signal, synaptic, and sensing systems. Hydrogel, as a gate dielectric, is the key factor for ionotropic devices owing to the excellent stability, ultra-high linearity, and extremely low operating voltage of the biodegradable and biocompatible polymers. Moreover, hydrogel exhibits ionotronic functions through a hybrid circuit of mobile ions and mobile electrons that can easily interface between machines and humans. To determine the high-efficiency neuromorphic chips, the development of synaptic devices based on organic field effect transistors (OFETs) with ultra-low power dissipation and very large-scale integration, including bio-friendly devices, is needed. This review highlights the latest advancements in neuromorphic computing by exploring synaptic transistor developments. Here, we focus on hydrogel-based ionic-gated three-terminal (3T) synaptic devices, their essential components, and their working principle, and summarize the essential neurodegenerative applications published recently. In addition, because hydrogel-gated FETs are the crucial members of neuromorphic devices in terms of cutting-edge synaptic progress and performances, the review will also summarize the biodegradable and biocompatible polymers with which such devices can be implemented. It is expected that neuromorphic devices might provide potential solutions for the future generation of interactive sensation, memory, and computation to facilitate the development of multimodal, large-scale, ultralow-power intelligent systems.

## 1. Introduction

Presently, massive information and data storage is needed for the remarkable development of information technology and artificial intelligence (AI), big data, cloud computing, and the Internet of Things (IoT). Consequently, large numbers of digital components are required to process and communicate suitably within digital computers based on conventional von Neumann architecture [1,2,3,4]. The physical separation of memory and control units of conventional digital computers is based on von Neumann architecture, and a large amount of energy is consumed in the processing of artificial intelligence, big data, cloud computing, and the Internet of Things [2,3,4,5,6,7]. As a result, integrating the computing requirements with both exceptional performance and minimal energy consumption proves to be a challenging task.

To solve this issue of complex and unstructured information competently, a new type of computing design and fabrication is required [4,6,7]. In contrast, the human brain serves as an intelligent information processing system, overseeing aspects of human perception, cognition, learning, and memory [3,4,5,6,7] with a tiny power consumption of ~20 W [3] as compared to conventional computer systems to execute cognitive tasks [6,8]. Inspired by the energy-efficient, robust, plastic, and fault-tolerant human brain, neuromorphic computing aims to attain high-performance computing through the emulation of neural and synaptic mechanisms [7,8,9,10,11,12]. The human brain, a crucial central nervous system component, comprises approximately 10^11^ neurons interconnected through 10^15^ synapses [1,2,3]. This intricate neural architecture facilitates communication essential for information processing and storage. The brain’s powerful capabilities, including neurotransmitter functions [5], enable it to address complex and unstructured information processing, contributing significantly to human perception, learning, thinking, and memory. Therefore, numerous research works on single solid-state neuromorphic devices have recently been developed to mimic the biological signal, including synaptic response and cognitive tasks, for artificial neuromorphic platforms [9,11].

The idea of incorporating neuromorphic devices with the capability to replicate synaptic and neural functions at the hardware level is exceptionally fascinating. Initially, synaptic functions are mimicked using complementary metal-oxide semiconductor (CMOS) neuromorphic circuits. However, these CMOS circuits consumed significantly more energy than a biological synapse, and scaling up the circuits to a size comparable to the brain posed considerable challenges. For implementation at the hardware level, various types of neuromorphic devices, such as two-terminal devices [6] and 3T devices, have been proposed as potential foundational components for synaptic bionics and applications in neuromorphic engineering, including phase change memory (PCM), resistive random access memory (RRAM) [13,14,15,16,17], magnetoresistive access memory (MRAM) [18], and so on, while 3T devices include floating-gate transistor devices [19,20], ferroelectric field effect transistors (FeFETs) [21], electrolyte-gated transistors (EGTs) [22,23,24,25,26,27,28], and hydrogel-gated field effect transistors (HGFETs) [29,30,31,32]. Due to the physical separation of the pre- and postsynaptic terminal, the 3T device is the best choice to simulate various neural activities, such as short-term plasticity (STP) and long-term plasticity (LTP), excitatory postsynaptic current (EPSC), and spike-timing dependent plasticity (STDP) [19,20,21,22,23,24,25,26,27,28,29]. Hydrogel-gated field-effect transistors (HGFETs), among the diverse array of 3T devices, have been explored for widespread practical application in bioelectronics owing to their resemblance to biological synapses [12,29]. In hydrogel-electrolyte-gated field-effect transistors, electrical double layers (EDLs) are formed due to the presence of numerous hydroxyl groups [3,33,34,35,36,37,38] at the interface between the hydrogel electrolyte and the channel. This formation results in the development of capacitance, leading to increased coupling productivity and demonstrating a low threshold voltage (on the order of millivolts) [39]. Owing to this reason, hydrogel-electrolyte-gated field-effect transistors are efficient low-power neuromorphic devices. Cross-linking the hydrophilic properties of hydrogels, which have a high water content akin to human tissues, results in a combination of ionic and electronic nature and has served as a soft bridge between electronic devices and human tissues to mimic biological signal exchange [2,40,41,42,43,44,45,46].

Regrettably, the majority of neuromorphic devices reported thus far are not eco-friendly and, in fact, can be harmful or toxic, i.e., their lack of biocompatibility renders them incompatible for application in human tissues or skin [33,40]. Moreover, the presence of harmful ingredients in these neuromorphic devices carries a major risk of environmental pollution, necessitating substantial resources for electronic waste management. Hence, there is a necessity for the incorporation of biocompatible or biodegradable materials in electronic product development [2,3,24,29,31,38,40,47,48,49].

**Figure 1:**
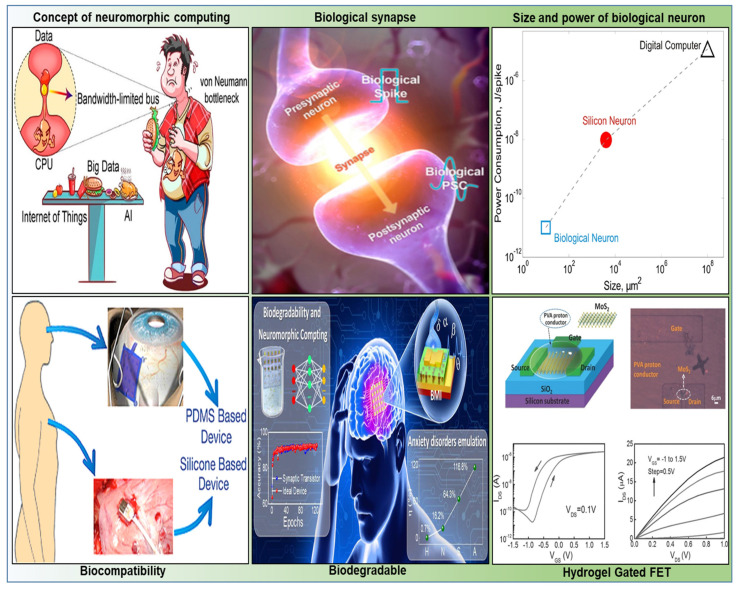
Concept of neuromorphic computing, Copyright 2021, American Chemical Society [7]. Biological synapse, Copyright 2021, American Chemical Society [1]. Biological neurons and digital computers in terms of power and space efficiencies, Copyright 2011, Frontiers in neuroscience [8]. Hydrogel gated FET: Schematic picture of the PVA hydogel gated 2D MoS_2_ synaptic transistor, Copyright 2017, Wiley Online Library [11]. Biodegradable: Biodegradable ITO based neuromorphic transistor, Copyright 2023, American Chemical Society [3]. Biocompatibility: Non-toxicity and device was transferred onto a human body, Copyright 2016, American Chemical Society [44]. Synaptic devices are discussed based on four diverse types of research areas.

A dedicated exploration into environmentally friendly materials is underway in response to the increasing importance of bio-functional electronic devices. Specifically, a range of organic materials, including hydrogel polyvinyl alcohol (PVA), milk, chitosan (CS), egg, albumin, and cellulose, has been utilized as electrolytes in making electrochemical double-layer (EDL) synaptic transistors [2,3,24,29,39,40]. These categories of transistors demonstrate characteristics such as biodegradability, non-toxicity, biocompatibility, and suitability for bio-friendly electronic systems and are sourced from abundant natural reservoirs, streamlining cost-effective processing [2,3,29,40,41,42,47,48,49,50,51]. Overall, Figure 1 clearly shows neuromorphic computing, including its structures, biodegradability, and biocompatibility. This review presents the recent progress and development of HGFETs in terms of neuromorphic applications. We briefly introduce the working principle and structure of an artificial synaptic transistor based on hydrogel-gated FET with 2D-channel materials. In addition to its biodegradability and biocompatibility, important problems, opportunities, future challenges, and solutions related to flexible artificial perception systems on essential applications in neurodegenerative diseases are also discussed.

## 2. Three-Terminal Organic Synaptic Transistor

**Figure 2:**
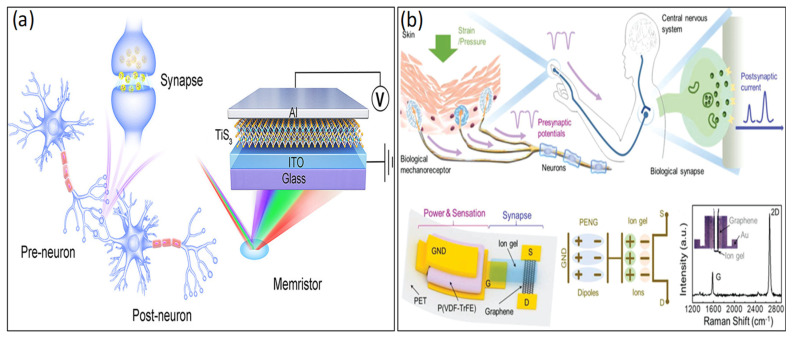
(**a**) Two-terminal and (**b**) three-terminal synaptic devices for an artificial neuron that consists of the dendrites, soma (which comprises the neuronal membrane and the spike event generation mechanism), and axon as the input, soma, and output, respectively. Copyright 2021, American Chemical Society [17]. Copyright 2019, Wiley Online Library [33].

To emulate ion-dynamics-based biological synapses that are flexible, inexpensive, and lightweight, an organic synaptic transistor stands out as the most suitable device choice that has recently been a growing development in the use of 3T transistors in synaptic simulation techniques [22,23,24,52,53]. Additionally, transistors offer precise device selection through low operating voltage, effectively addressing adjacent cell crosstalk in two-terminal synaptic electronic devices [29,30,31,32,33,34,35,36,37,38,39]. Moreover, 3T-based synaptic transistors have more consistent current conduction through the channel. They are stable in changes in the gate voltage as compared to two-terminal memristor devices because of nonlinear current conduction through the oxide filament [12,39,40]. To emulate bio-synaptic functions, a range of synaptic device configurations has been suggested, including two-terminal (2T) and three-terminal (3T) synaptic devices, as demonstrated in Figure 2a,b.

In terms of organic artificial synapses, transistors exhibit significant potential for upcoming neuromorphic electronics due to their benefits, including (1) compatibility with solution-printing and micro-patterning lithography processes; (2) the straightforward adjustability of molecular, chemical, electrical, and mechanical properties to serve various purposes; and (3) mechanical flexibility and stretchability, exhibiting a low elastic modulus similar to that of biological neurons [3,4,24]. Furthermore, considering the impact of device size, which significantly influences energy consumption (E), organic materials can be structured into submicron dimensions through cost-effective methods like solution printing or photolithography. The microminiaturized organic synapses thus produced have the potential to achieve low energy consumption. Utilizing organic artificial synapses driven by field-induced ion migration can generate responsive and adaptive synaptic reactions to subtle stimuli, resembling the neurotransmitter responses observed in biological synapses [1,2,3]. Consequently, the application of these energy-efficient organic artificial synapses holds promise for the development of brain-inspired computing, memory systems, AI, biomimetic robotic sensory, and motor nervous systems, as well as neural prostheses that align with the efficiency of natural biological processes. However, implementing CMOS technology poses challenges that necessitate resolution, such as addressing the instability of n-type materials, managing the mismatch in carrier mobility (µ) and threshold voltage (V_th_) between n- and p-type devices, and navigating through intricate manufacturing processes [24,33]. At the transistor level of sensing components, additional reductions in energy consumption (E) can be achieved through strategies such as downsizing device dimensions, minimizing the energy barrier between organic semiconductors and source/drain electrodes, and employing a gate insulator with a high dielectric constant, along with organic semiconductors characterized by high carrier mobility (µ).

The synaptic function, materials, and structures of organic synaptic devices and their working mechanisms and biodegradable and biocompatibility are briefly discussed in this section.

### 2.1. Neuronal and Synaptic Functions Mimicked by Organic FET

The biological neural network processes information by means of synaptic activities. Presynaptic neurons generate nerve impulses (action potentials) upon receiving input (stimulus information), which are then transmitted to postsynaptic neurons through the synapses [3,5,8,9,10,11,12], resulting in postsynaptic currents. In general, the postsynaptic currents range from several hundred picoamperes (pA) to a few tens of nanoamperes (nA) [1,2,3,4,5,6,7]. Hence, synapse is the key factor to connect neurons, and it can execute processing function and information storage over the synaptic weight [7,10,13]. A change in synaptic weight is synaptic plasticity, confirming the neurotransmitters are released into the cleft from the presynaptic terminal and attracted to the postsynaptic terminal [10,11]. The action potential induces depolarization of the cell membrane, leading to the opening of voltage-gated Ca^2+^ channels. This allows Ca^2+^ ions to enter the presynaptic membrane due to concentration gradients. The entry of Ca^2+^ ions into the cytoplasm triggers the release of neurotransmitters (specialized proteins for interneuronal communication) into the synaptic cleft [2,3,7]. These neurotransmitters subsequently bind to the receptors on the postsynaptic neuron, activating the ligand-gated channel for Na^+^ ions to enter the postsynaptic membrane. This process facilitates transmitting and communicating the input stimulus information to the adjacent neuron [1,2,3,4,5,6,7,54,55,56,57]. The attachment of neurotransmitters to the receptors on the postsynaptic membrane alters the shape of these receptors, initiating a sequence of reactions that lead to the opening of ion channels.

**Figure 3:**
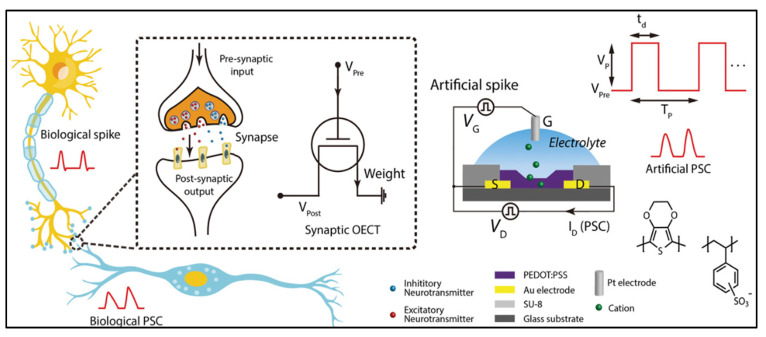
Schematic diagram of a biological synapse with applied action potential and the equivalent 3T synapse, the sketch of a solution-gated PEODT:PSS OECT, and molecular structure of PEDOT:PSS. Copyright 2019, Wiley Online Library [12].

**Figure 4:**
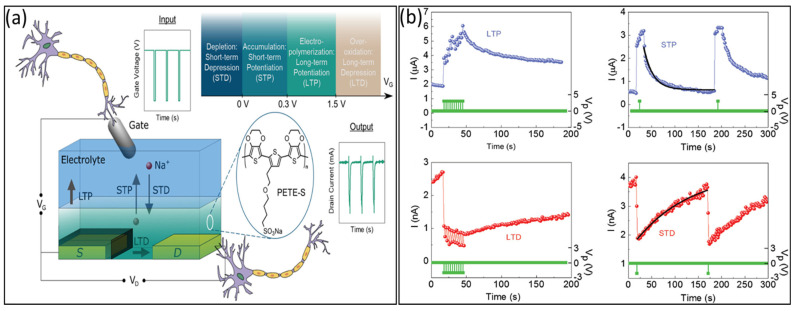
(**a**) Mimicking behavior in the human brain of plasticity mechanism. Copyright 2019, Wiley Online Library [55]. (**b**) Synaptic performance of LTP, STP, EPSC, and IPSC. Copyright 2019, Wiley Online Library [56].

A schematic diagram of biological and artificial 3T synapses is presented in Figure 3, comprising three essential components: the presynaptic neuron, the synaptic cleft, and the postsynaptic neuron. In the realm of biological synapses, information transmission and processing are intricate processes [57]. The connection strength between neurons, represented by the synaptic weight (W), is contingent on the concentrations of ionic species (e.g., Ca^+^, Na^+^, K^+^, and Cl^−^) triggered by presynaptic action potentials, effectively regulating the release of neurotransmitters [40,41,42,57]; essentially, this refers to the memory effect that occurs following transmission. Synaptic plasticity, the ability of the synaptic weight (representing the connection strength between neurons), can be altered by either one or both sides of the synapse, enabling dynamic modifications in neural communication [41], which is influenced by the characteristics of action potential pulses/spikes [1,3,5]. Synaptic plasticity can be classified into two categories based on retention time: short-term plasticity (STP) and long-term plasticity (LTP). STP, which lasts only milliseconds to minutes and sustains long-term, while LTP is closely associated with memory function in the human brain, plays crucial roles in shaping neural information processing, and contributes to the dynamic modulation of neural circuits during various cognitive functions [55,56]. In addition, the synapses can be categorized into two types based on receptors on the postsynaptic membrane, excitatory and inhibitory. The excitatory synapses induce excitatory postsynaptic current (EPSC), stimulating the postsynaptic neuron to initiate an action potential [33,41,57]. Figure 4a,b illustrates the mimicking behavior in the human brain using organic artificial synaptic transistors. On the other hand, at inhibitory synapses, the inhibitory postsynaptic current (IPSC) is produced, pushing the postsynaptic neuron away from the threshold required to generate an action potential [41] for mimicking the biological signal. Hence, the processing of complex information is predominantly executed through the fundamental neural activities of EPSCs and IPSCs by applying a voltage pulse at the gate terminal (presynaptic). Investigating the physical mechanism of artificial synaptic devices under electrical modulation involves analyzing the influence of relative positive/negative control gate voltage pulses on channel currents to comprehend the behavior of IPSCs and EPSCs [1,3,24]. The source–drain current of the organic synaptic transistor exhibited stability prior to the application of the presynaptic pulse. Upon exposure to relative positive or negative voltage pulses at the gate terminal, the source–drain current experienced enhancement (EPSC) or suppression (IPSC) accordingly. It is important to highlight that the current does not exhibit a monotonic return to the initial level even after the pulse is removed. Conversely, when the positive (or negative) voltage pulse at the gate terminal reverts to the initial level, the source–drain current demonstrates a postsynaptic current behavior, characterized by a slow relaxation phenomenon.

### 2.2. Materials of Organic Synaptic Transistors

Addressing the energy consumption challenge is crucial in neuromorphic electronics, where processing vast amounts of intricate data surpasses the confines of traditional von Neumann computing. To enhance energy efficiency in neuromorphic systems to match the remarkable levels found in biological nervous systems, the human brain serves as a compelling model, consuming 1–10 fJ for each synaptic event, while synthetic synapses are subject to diverse factors [55,56,57,58]. To achieve this for an artificial synapse, modifying the device dimensions, materials, and mechanisms employed in synaptic transistors is crucial, as energy is a product of voltage (V), integration current (I), and time (t). To obtain a reduction in current (I), it is imperative to minimize the device dimensions by significantly decreasing the cross-sectional area (channel width) through which the current flows [3,42,55,57]. Likewise, to diminish the spike duration (t), the channel thickness can be reduced, or the diffusion coefficient can be increased as ions within the electrolytes move and interact with the channel layers upon applying a gate voltage [56,57,58,59,60]. Various organic materials used in artificial synaptic transistors, such as channel materials, gate electrolytes, device structure, and energy efficiency in neuromorphic systems, are summarized in Table 1 in this paper. 

In this section, we unveil a variety of distinctive materials serving as active layers (channel materials), organic semiconductors, redox electrochemical organic semiconductors, and composite materials based on biocompatibility, biodegradability, and gate electrolyte. Additionally, significant attention is given to other materials, such as liquid ion electrolytes and solid ion gate electrolytes, all utilized as dielectric layer materials.

#### 2.2.1. Channel Materials

**Figure 5:**
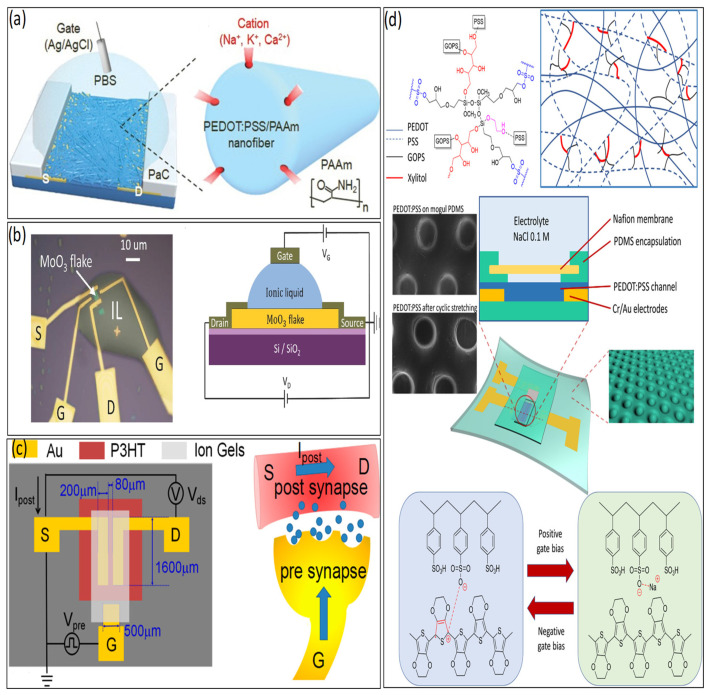
(**a**) Schematic of a channel using PEDOT:PSS/PAAm nanofiber. Copyright 2021, Wiley Online Library [60]. (**b**) Channel with MoO_3_. Copyright 2017, Wiley Online Library [61]. (**c**) Channel with P3HT-based low power synaptic transistor. Copyright 2016, American Chemical Society [62]. (**d**) Schematics of biological synapses and neurotransmitter-mediated PEDOT:PSS channel modified with GOPS upon thermal annealing, PEDOT:PSS and NaCl electrolyte, de-doping and doping processes of PEDOT:PSS under positive and negative gate biasing. Copyright 2021, Wiley Online Library [26].

Various organic synaptic devices, using diverse semiconductor materials, can mimic synaptic neurons with various functionalities as low-cost chips. Organic materials are used as channel materials for the synaptic transistors to minimize the device size mentioned in Figure 5. The atomic structure of 2D materials (such as graphene and transition metal chalcogenides) offers high integration, reduced short-channel effects, and low leakage current conductions [2,3,29]. After applying a positive gate voltage to the gate electrode, the electrolyte ions (protons) enter into the transistor channel [1,2,3,17]. For example, in 2021, Lee et al. fabricated a synaptic transistor with PEDOTS:PSS/PAAm organic materials as channels holding Na^+^, Ca^+^, and K^+^ cations and accomplished low switching energy of nearly 113 fJ, without the loss of contingent information [60], as shown in Figure 5a. In 2017, Yang et al. also fabricated organic transistors with MoO_3_ as channel materials, with an energy consumption of 9.6 pJ per synaptic event [61], as shown in Figure 5b. In 2016, Qian et al. fabricated and demonstrated organic, flexible transistors by poly(3-hexylthiophene (P3HT) as semiconductor channel materials for low-power synaptic devices per synaptic event [62], as shown in Figure 5c. In 2021, Tung et al. fabricated PEDOT:PSS and asserted that when a positive gate voltage is applied, protons within the biomaterial are driven, leading to a de-doping electrochemical reaction. Conversely, a doping reaction occurs when a negative gate voltage is applied [20]. In 2022, Trong et al. demonstrated the modified PEDOT:PSS channel based on stretchable OECTs. The de-doping and doping procedures of PEDOT:PSS when subjected to positive and negative gate bias were applied. It is a stable channel material that is tunable and can maintain the device’s performance. Additionally, it is easy to dissolve in water, as shown in Figure 5d [26], indicating biodegradable and biocompatible materials. However, there needs to be more research on device scalability, operating speed, and channel materials, necessitating further studies to be conducted in these areas.

#### 2.2.2. Gate Electrolyte

By utilizing ionic compounds as the gating media, ion-gated transistors (IGTs) play a crucial role in advancing neuromorphic computing devices, primarily due to their ability to operate at low voltages and be scalable for fabrication into large device arrays [61,62,63,64]. Due to their biocompatibility, biodegradability, and mechanical similarity to human skin, ionic gels show great potential as soft conductors for E-skins, implantable biosensors, and wearable biosensors in the realm of human health monitoring [2,3,24,29,31,49,65].

**Figure 6:**
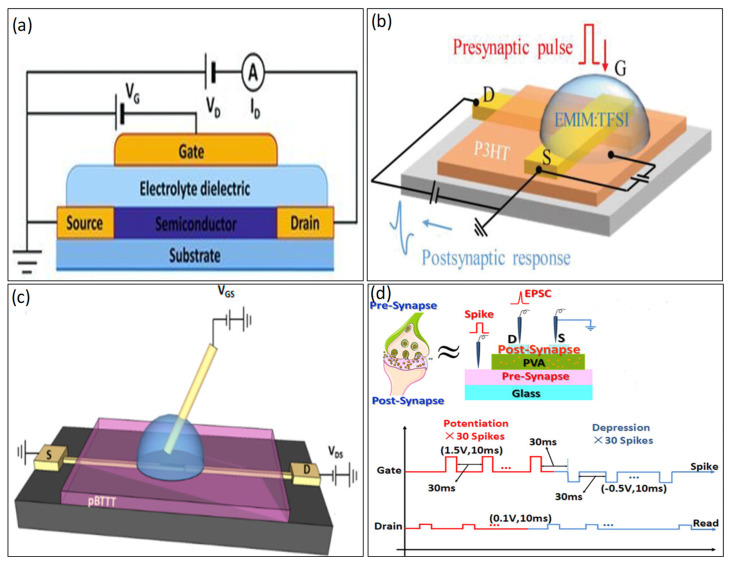
(**a**) Cross-section of an 1-ethyl-3-methylimidazolium bis(trifl uoromethylsufonyl)vamide ([EMI][TFSA]) as an electrolyte-gated transistor. Copyright 2013, Wiley Online Library [19]. (**b**) Ionic liquid 1-ethyl-3-methylimidazolium bis(trifluoromethylsulfonyl)imide (EMIM:TFSI) ionic-gated synaptic transistor. Copyright 2022, Wiley Online Library [67]. (**c**) Scheme of the PBTTT-C16 PAA-based hydrogel synaptic transistor. Copyright 2018, American Chemical Society [72]. (**d**) PVA-gated neuromorphic transistor, Copyright 2023, American Chemical Society [24].

In 2013, Lee et al. displayed the first ionic-liquid-based gel organic transistor [19], as shown in Figure 6a. Presently, various types of ion-gels are used as a dielectric medium. For example, 1-ethyl-3-methylimidazolium bis (trifluoromethylsulfonyl) imide [EMIM][TFSI] [66,67,68,69], [1-ethyl-3-methylimidazolium bis(trifluoromethylsulfonyl) imide [EMIM][TFSA] [19,70,71], and 1-butyl-3-methylimidazolium hexafluorophosphate [BMIM][PF6] [72], have been fabricated and are widely used and show favorable performance. In 2009, Lee et al. [EMIM][TFSI] used as a gate dielectric, which was remarkably large (>10 µF/cm^2^ at 10 Hz) and had low operating voltage (<2.5 V) [67]. In 2022, [EMIM][TFSI] was used as an ion-gel electrode for synaptic transistors by Liu et al. [67], as presented in Figure 6b. The remarkable combination of short channel length and an immensely large capacitance in the electric double layer at the electrolyte–channel interface results in an impressively low power consumption of 0.06 fJ per synaptic event, far surpassing the requirements of biological synapses (1–10 fJ) [68].

Hence, most ion-gel is not fully biocompatible and biodegradable [24,68] in nature. Instead of ion-gel, hydrogels, much like human tissue, are primarily water-based, promising candidates for biomimetic materials owing to ample water absorption and fast ion transport capability through 3D networks. However, dealing with their distinct strain-stiffening properties and high stiffness simultaneously remains a vital hurdle to overcome. The key merit of E-skin is self-healing, which, in hydrogels, offers advantages in terms of longevity, mechanical durability, and dependability [72]. In Figure 6c, Fillaud et al. demonstrated switchable PBTTT-C16 PAA-based hydrogel-gated field-effect synaptic transistors and created a novel category of adaptable, low-power, and exceptionally sensitive biosensors capable of detecting substances in aqueous environments [72]. Proton-conducting electrolytes, hydrogel poly (vinyl alcohol) (PVA) used as gate dielectrics, were fabricated into synaptic transistors by Guo et al. in 2019 [24], as shown in Figure 6d. PVA-gated neuromorphic devices display crucial characteristics of ultralow energy consumption, approximately 1.16 fJ, and ultrahigh sensitivity (5.4 dB), making them highly valuable for applications in neuromorphic computing.

### 2.3. Significant Type of Artificial Synaptic Transistor

Over the last few decades, SiO_2_-based gate dielectric field effect transistors have been widely used, although they exhibit a comparatively low capacitance due to the limited level of carrier accumulation charges experienced by applying gate voltage [22,23,24,25,26,27,28,71,72,73]. Being that they are soft and tunable and have high dielectric capacitance due to the production of EDL in the dielectric and channel region, organic field effect transistors (OFET) represent the materials that facilitate the coupling and transportation of both electronic and ionic charges [24]. In flexible synaptic devices based on 3T transistors, the gate electrodes function as presynaptic terminals, while the channel layers are considered postsynaptic membranes. Flexible synaptic devices based on 3T transistors transmit signals through their channel layer and independently modulate synaptic weight via the gate terminals. Hence, these devices can address the limitations inherent in 2T-based synaptic devices when emulating natural synapses, opening up various potential applications in brain-inspired computing and artificial neuromorphic systems.

In general, 3T-based flexible synaptic devices can be categorized into the following three main types based on their distinct operational principles.

#### 2.3.1. Floating-Gate Synaptic Transistor

**Figure 7:**
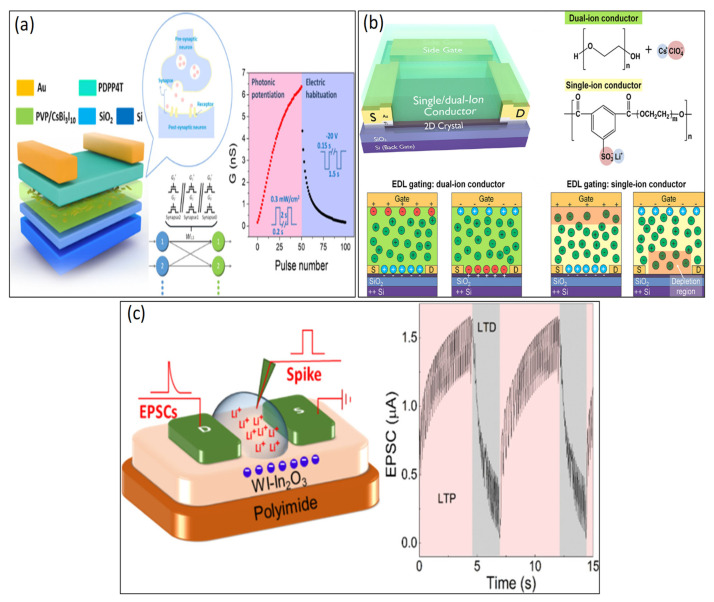
(**a**) Floating gate FET. Copyright 2022, IOP Science [73]. (**b**) ELDT. Copyright 2019, American Chemical Society [79]. (**c**) ECT: pre-and postsynaptic layers are separated by an electrolyte layer transporting ions/protons, Copyright 2019, American Chemical Society [80].

Synaptic devices based on floating-gate transistors (FGTs) typically feature a device structure similar to conventional FETs, with the distinction that the control gate in FGT is embedded within the dielectric layer [73], as shown in Figure 7a. Applying a gate voltage enables electronic charges to be effortlessly injected into the floating gate through thermal emission or quantum tunneling, facilitating storage owing to the presence of robust charge blocking and tunneling layers [70,71,72,73,74,75]. The threshold voltage *V_t_* of the FGT is expressed below in Equation (1):

(1) $$V_{t}=K-\frac{Q_{fg}}{C_{cg}}$$

where *Q_fg_* and *C_cg_* represent the floating gate charge and the capacitance between the control gate (*C_g_*) and floating gate (*F_g_*), respectively. *K* is a device manufacturing process constant.

Applying a writing-gate voltage allows charges in the channel to traverse the tunneling layer using either Fowler–Nordheim (F–N) tunneling or a channel hot electron injection mechanism, enabling their storage in the floating gate layer. Consequently, the *Q_fg_* can modify the device’s threshold voltage during a write/erase operation.

In 2018, Ren et al. introduced a flexible synaptic transistor featuring a low operational voltage and controlled charge-trapping polarity by employing C60/PMMA as the hybrid layers for tunneling and floating-gate functionalities [74]. Wang et al. demonstrated the successful creation of organic thin-film transistor memory devices through the incorporation of a layer of nanoparticles (e.g., Ag or CaF_2_) positioned between two nylon six-gate dielectrics, acting as the floating gate [76]. Qu et al. designed and fabricated a flexible sensory memory device utilizing a carbon nanotube thin film as the active channel and a controllably oxidized aluminum nanoparticle array as the floating gate [77]. Remarkably, this device achieved a high on–off current ratio and displayed long-term retention for both electrical and optical programming capabilities [78]. Flexible synaptic devices based on floating-gate transistors (FGTs) offer significant benefits, including vast memory capacity and cutting-edge fabrication technology [79]. Despite these advantages, there are still some challenges to address, such as high operation voltage and the absence of long-term synaptic plasticity [74,75,76,77,78], which require resolution.

#### 2.3.2. Electric-Double-Layer Synaptic FET (EDLST)

The electric-double-layer synaptic FET (EDLST) is a highly promising device within flexible synaptic devices. Through a capacitive field-effect mechanism at the channel/electrolyte interface (Figure 7b), the gate voltage modulates the channel current in the device.

By employing electrolytes, positive/negative charges will accumulate at the interface between the electrolyte and semiconductor. This interface consists of electrons (holes) within the semiconductor and cations (anions) in the electrolyte. Consequently, novel synaptic transistors based on electric-double-layer transistors (EDLTs) emerge, showcasing significantly enhanced carrier density attributable to the establishment of an electric double layer (EDL) at the semiconductor/electrolyte interface.

The relationship between the accumulated charge density (*Q*), specific capacitance (*C_s_*), and the voltage applied to the gate electrode (*V_g_*) in FET can be expressed by the following equation:

(2) $$Q=C_{s}V_{g}$$

Hence, the drain current equation of the FET can be written as

(3) $$I_{ds}=\mu C_{s}\frac{W}{L}\left[\left(V_{g}-V_{t}\right)V_{d}-V_{d}^{2}\right]$$

where *I_ds_* is the channel current; *μ* is the hole mobility; *C_s_* is the specific capacitance; *V_g_* is the gate voltage; *V_d_* is the drain voltage; *V_t_* is the threshold voltage; and *W* and *L* are the width and length of the channel, respectively.

Equations (2) and (3) illustrate that an increased capacitance results in a greater density of accumulated carriers, a reduced gate switching voltage, and a heightened on-current density [80,81,82]. As a result, the EDLT-based flexible synaptic devices achieve ultralow operation voltages in the millivolt range, which is attributed to their exceptional coupling efficiency from the gate to the channel [80]. This characteristic makes them an appealing option for energy-efficient neuromorphic circuits [80,81].

A series of investigations on EDLT-based flexible synaptic devices was carried out by Wan’s research group [81]. They developed flexible planar gate synaptic transistors using a polymer electrolyte as the gate dielectric material, successfully accomplishing multiple synaptic functions in their research [82,83,84,85,86,87,88,89,90]. They introduced a laterally coupled oxide-based EDLT, enabling spike logic operation and modulation by incorporating multiple presynaptic inputs. In another research endeavor, in 2016, Wan et al. utilized a proton-conducting GO film as the electrostatic coupling electrolyte in multigate oxide-based neuron transistors [81]. These devices effectively emulated paired-pulse facilitation, dendritic integration, and orientation tuning functions.

Nonetheless, the presence of a liquid electrolyte in these devices poses compatibility challenges with photolithography technology, making it difficult to achieve a large area and uniform preparation of the devices.

#### 2.3.3. Organic Electrochemical Synaptic FETs (OECSFETs)

Flexible synaptic devices based on organic electrochemical synaptic FETs represent another category of devices that effectively utilize the ionic species within the redox-active channel material [3,29,30,31,32,73,91,92,93,94,95]. These devices rely on electrochemical doping/restoration processes achieved by bulk injection of ionic species, which, in turn, modulates the channel conductivity via the electrolyte dielectric layer (Figure 7c) [95]. Accordingly, the Bernards model heavily relies on the analogy with MOSFET behavior, as described by Daniel Bernards and George Malliaras [92]. It employs the same set of equations used for charge transport in long-channel MOSFETs [4], resulting in a comparable description of electronic charge transport within the organic electrochemical synaptic FETs channel:

(4) $$I_{ch}=\left\{\begin{array}{c} \mu C^{*}\frac{Wd}{L}\left[1-\frac{V_{g}-\frac{1}{2}V_{d}}{V_{t}}\right]V_{d}\;for\;V_{d}>V_{g}-V_{t}\\ -\mu C^{*}\frac{Wd}{2L}\frac{{\left[V_{g}-V_{t}\right]}^{2}}{V_{t}}\;for\;V_{d}<V_{g}-V_{t} \end{array}\right\}$$

where *I_ch_* is the channel current; *μ* is the hole mobility; C* is the volumetric capacitance; *V_g_* is the gate voltage; *V_d_* is the drain voltage; *V_t_* is the threshold voltage; and *W*, *d*, and *L* are the width, thickness, and length of the channel, respectively.

Deriving from Equation (4) above, the transconductance (g_m_) of an OECSFETs can be calculated. Transconductance, defined as the derivative of the channel current concerning the gate voltage, characterizes the OECSFETs’ ability to amplify a voltage signal applied to the gate electrode. This parameter holds significance in various applications, including biosensing, digital logic, and neuromorphic engineering, where OECSFETs are configured to detect the input voltage at the gate electrode (*V_g_*) by measuring its impact on the output current through the OECSFETs channel (*I_ch_*).

The drain current, represented as *I_ds_*(*t*), results from a combination of the displacement current and the channel current, weighted accordingly. Bernards and Malliaras performed calculations for *I_ds_*(*t*) under the condition of a square gate voltage step, demonstrating that *I_ds_*(*t*) exhibits an exponential time dependence described by the following:

(5) $$I_{ds\;}\left(t\right)=I_{ds}\left(V_{g}\right)+∆I_{ds}[1-f\frac{\tau_{e}}{\tau_{i}}]e^{\frac{-t}{\tau_{i}}}$$

where *I_ds_* (*t*) at time t, *I_ds_* (*V_g_*) saturation drain current at *V_g_*, ∆*I_ds_* [∆*I_ds_* = *I_ds_*(*V_g_* = 0) − *I_ds_*(*V_g_*)] is the current difference between initial and final steady state current, *f* is the weighting factor, and *τ_e_* and *τ_i_* are the electronic transit time along with channel and time constant, respectively.

Equation (5) indicates the existence of two distinct qualitative regimes in transient behavior, monotonic relaxation (when the electronic transport exceeds the speed of ionic charging) and spike and recovery (when the ionic charging exceeds the speed of electronic transport), for an OECSFET due to the channel geometry. The device performance will be slow for larger channel geometry. To enable the low-power operation of the transistor in the two distinct characteristic regimes above, Bernards and Malliaras adjusted *V_d_* to manipulate this ratio [4,92].

In 2023, Oh et al. demonstrated an electrochemical neuromorphic organic device fabricated on flexible substrates. The device architecture involved a postsynaptic electrode (PEI/PEDOT:PSS film) interfacing with a presynaptic electrode (PEDOT:PSS) through an electrolyte [89]. Notably, this device displayed a vast array of nonvolatile and reproducible states, exceeding 500, while operating at remarkably low operating voltages [91]. In 2018, Yang and colleagues developed an artificial synaptic transistor based on flexible tunable gate memristive behavior by employing a deposition process involving SWCNTs and polymer composites onto a hydrophilic dielectric layer [96].

The application of an electric field to the device enabled control over the electrochemical potential difference between SWCNT and H_2_O/O_2_ redox couples, consequently influencing the channel current. This flexible artificial synapse outperformed existing devices in multiple aspects, including energy consumption, device dimensions, state capacity, and linearity. The ECSFET offers persistent control over channel conductance without the need for power, making it exceptionally beneficial for electronic synapses in neuromorphic chips [97,98,99]. However, research limitations concerning device scalability, operating speed, and electrolyte stability exist. Further studies are necessary to address these aspects and advance neuromorphic technology.

#### 2.3.4. Biodegradability and Biocompatibility

Research on biodegradable and biocompatible ECT-based synaptic devices has been tremendously concentrated on mitigating the environmental contamination stemming from electronic waste. It is used in medical devices, implants, tissue engineering, and drug delivery systems to ensure safe and effective interactions with the human body, respectively [76,86,91,97]. This section is focused on reviewing the biocompatibility and biodegradability of ECT synaptic transistors. Natural biomaterials exhibit the potential to create electronic devices that are both environmentally degradable and enable a wide range of applications, like future eco-friendly data storage solutions [99]. Soft and stretchable bioelectronics for digital healthcare, which has garnered significant research attention, rely on the presence of biocompatible conductors as crucial components [99,100,101,102]. Compared to other polymers, natural biopolymers offer distinct advantages as potential building blocks for biocompatible conductors, including their excellent biodegradability and biocompatibility, abundant availability in nature, sustainability, and versatility for being processed into various functional formats with tunable material properties, all achieved under environmentally benign conditions.

**Figure 8:**
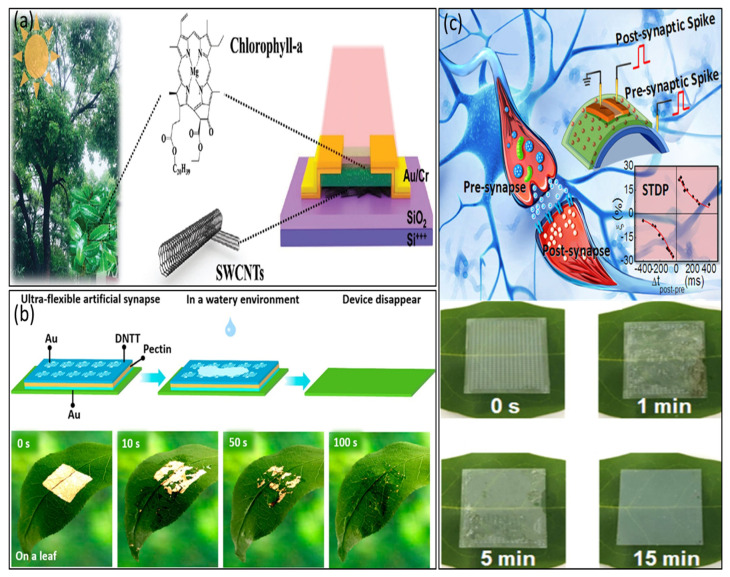
(**a**) Schematic of the chlorophyll-a/SWCNTs synaptic transistor. Copyright 2021, Wiley Online Library [100]. (**b**) Visual diagram of devices degrading in an environment, and degradation of the device caused by water on a leaf. Copyright 2022, American Chemical Society [101]. (**c**) Biodegradable property in air of chitosan/ITO interface synaptic transistor arrays. Copyright 2018, American Chemical Society [46].

In the year 2020, Yang et al. [99] made groundbreaking advancements by integrating natural chlorophyll into organic semiconductors, resulting in the development of a multifunctional organic transistor capable of accomplishing both photodetection and synaptic functions through precise gate voltage control. While integrating natural chlorophyll opens up the potential for degradable synaptic transistors, the devices’ degradability remained unexplored due to their construction on rigid substrates. In 2021, Ou et al. extended their research efforts by combining chlorophylla and carbon nanotubes, leading to the creation of degradable photonic synaptic transistors, as illustrated in Figure 8a [100]. In Figure 8b, pectin is applied as an ion gel for synaptic transistors that showcases the environmentally sustainable characteristics of the transistor array, highlighting its impressive degradability and paving the way for the development of next-generation degradable neuromorphic electronics [101].

In 2022, Liu et al. demonstrated synaptic transistors that are both degradable and highly flexible by utilizing natural apple pectin as the dielectric material (Figure 8c) [46]. Impressively, in 2022, Li et al. successfully developed self-supporting organic synaptic transistors designed for neuromorphic applications that possess remarkable flexibility and degradability thanks to natural polysaccharide dielectrics [101]. The aforementioned research underscores the promise of environmentally sustainable electronics for advancing artificial neural networks, encompassing achievements ranging from the degradability of monolayer materials to the development of self-supporting devices with exceptional flexibility and degradable characteristics.

Nonetheless, it continues to face challenges that span from partial degradation to complete breakdown, and there is a need for research to develop fully degradable devices.

In terms of biocompatibility, ensuring it is a critical necessity when interfacing devices with the human body to prevent immune reactions and harm to biological cells to diminish foreign-body reactions [2,3,103,104,105,106,107]. As a result, extensive research has been carried out in the realm of devices utilizing biomaterials or harnessing biochemical signals. Electrolyte-gated synaptic transistors prove to be ideal devices for interfacing neuromorphic devices with biological neural networks [19,97,103,104]. Utilizing electrolytes as gates with high-k oxide, including liquid and ion gels, facilitates direct connections with living organisms has been analyzed [17,21,29,30,31,105]. The findings enable the detection and processing of biological information, paving the way for applications in biosignal sensors or neuroprostheses [106,107,108,109,110]. Incorporating biomaterials as electrolytes represents a viable approach to enhance the biocompatibility of these devices by applying natural biopolymers, including pectin [101] and protein-based [2] and thermo-responsive hydrogels [7,24], and shows excellent biocompatibility. Consequently, a device utilizing a solid-state gelatin–glycerol gel and polyvinyl alcohol (PVA) as the electrolyte was created for biocompatible devices [24,60]. This protein and PVA-based synaptic transistor employs protons as carriers, which function as neurotransmitters in biological systems.

**Figure 9:**
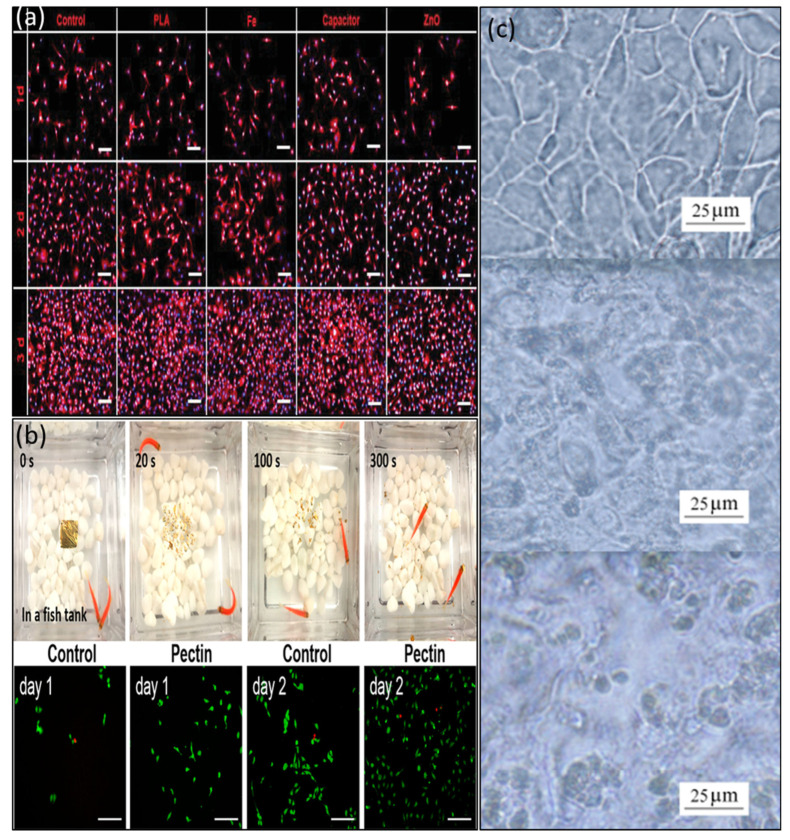
(**a**) Biocompatibility of PVA/PBS materials of BC for attachment, proliferation, and morphology of the L929 cells at different time scales. Copyright 2019, Wiley Online Library [49]. (**b**) Biocompatibility of the device confirmed by the vitality of the fish and fluorescent images of mouse-derived MC3T3 cells marked with calcein−AM (live cells, green fluorescence) and PI (dead cells, red fluorescence). Copyright 2022, American Chemical Society [101]. (**c**) Optical images of cancer cell lines cultured on PEDOT:PSS films (top to bottom) before and after being treated with retinoic acid in culture medium for 1 h and 7 h, respectively. Copyright 2010, Wiley Online Library [108].

In 2019, Li et al. designed and fabricated PVA/PBS hydrogel-gated devices for a bioabsorbable capacitor (BC) [49]. The cultured L929 cells showed high viability (>95%). From the 1st day to 3rd day, the L929 cells cultured displayed above 95% viability, indicating decent biocompatibility of the PVA/BPS materials of BC, as shown in Figure 9a. Natural pectin-based ion gel displays biocompatible materials for fabricating synaptic devices [101]. For the nontoxicity analysis, pectin-based organic synaptic devices were soaked in a fish tank, and after 300 s or a week under the same conditions, it was observed that the fish were still stable, suggesting nontoxic materials [101]. For the cytotoxicity test, a cell culture was performed using a pectin solution, and the cells still increased normally after 2 days, suggesting that the synaptic transistor was fully biocompatible and nontoxic, which was demonstrated by Li et al. in 2022 and is shown in Figure 9b. In 2010, Lin et al. claimed that PEDOT:PSS exhibits excellent biocompatibility with fibroblast cell lines (HFF1) [108]. After adding retinoic acid for one hour, a noticeable morphological transformation becomes apparent-nearly all cell lines on the device exhibit rupture and a lack of distinct boundaries between them. Subsequently, after 7 hours of treatment, most cells dissolve and detach from the device without any harm as displayed in Figure 9c.

However, there needs to be more research on device scalability, operational speed, electrolyte stability, biodegradability, and biocompatibility, indicating a need for further studies in these domains.

## 3. Artificial Intelligence for the Diagnosis of Neurodegenerative Disorders

When the functionality of nerve cells in the brain diminishes over time and ultimately fails, it is referred to as a neurodegenerative disorder [110,111,112,113]. To analyze a neurodegenerative disease, brain–machine interfaces (BMIs) are dynamic devices that link brains and machines for interpreting and transmitting neurological information. In BMI technology, mobile intelligent electronic devices and brain-inspired neuromorphic chips are of great interest in terms of neurodegenerative disease diagnosis. The synaptic weights between the neurons depend on the concentrations of ionic species under control in action potential, which is modulated by the neurotransmitter [112]. Neurodegenerative disease depends on the loss of synapses due to an abnormal release of neurotransmitters. Various mental and neurodegenerative disorders, such as schizophrenia, depression, and Parkinson’s disease, are associated with disruptions in dopamine transmission.

Cognitive neurodegenerative diseases, such as anxiety disorder and Alzheimer’s, are associated with abnormal amounts of amyloid beta (Aβ), and regulation for releasing the neurotransmitter acetylcholine is reported, resulting in significant synaptic loss in the connections between neurons essential for memory and learning [12,113]. Recently, researchers have suggested that anxiety, stress, and emotional shock may serve as initiates for Parkinson’s disease.

**Figure 10:**
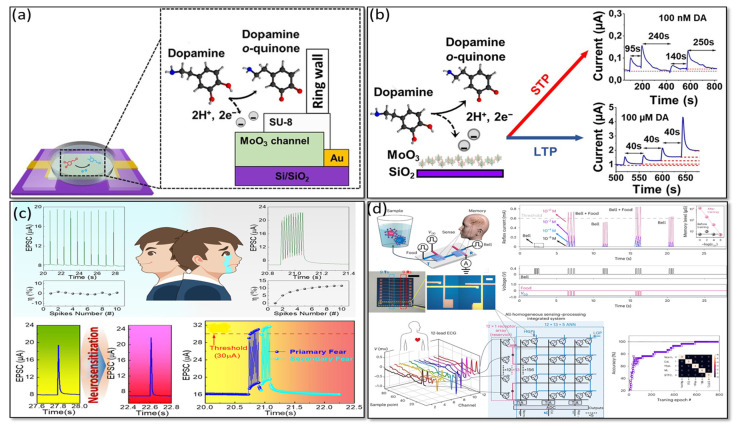
(**a**) Schematic diagram of a MoO_3_-based synaptic transistor with a projected dopamine oxidation mechanism. Copyright 2023, American Chemical Society [113]. (**b**) How dopamine influences the synaptic properties with respect to pulse signal. Copyright 2023, American Chemical Society [113]. (**c**) State of a healthy person and the neurosensitization process under external stimuli with the EPSC triggered by repeated presynaptic spikes, and response to the same spikes in a healthy state and after neurosensitization displayed “primary fear” and “secondary fear”, triggered by two spike trains (1 V, 10 ms) with a Δt of 20 ms. Copyright 2023, American Chemical Society [3]. (**d**) Schematic of the 1T1R learning unit that contains two identical cv-OECTs and simulated recognition accuracy of ECG waveform throughout. Copyright 2023, Nature [97].

In 2023, Tran et al. developed a biosensor synaptic transistor that could be capable of direct response to electrochemical stimuli through the release of dopamine neurotransmitters [113], as shown in Figure 10a,b. It is revealed that the dopamine-regulated plasticity tolerates the devices to control a specific range of dopamine concentration, from 100 nM to sub-mM levels. In 2023, Wang et al. presented a chemically facilitated artificial neuron (Ag NPs-silk fibroin/Ag memristor and PVA/SiO_2_/DA hydrogel-based temperature sensor) with the ability to both receive and release neurotransmitter dopamine while exhibiting synaptic plasticity [112]. Chen’s research group has claimed that an artificial neuron could transmit chemical information to an afferent nerve, establishing controlled initiation of motion in a mouse leg and robotic hand.

In 2022, Wang et al. demonstrated that anxiety disorder activities were emulated successfully, revealing “neurosensitization”, “primary and secondary fear”, and “fear adrenaline secretion-exacerbated fear” [3], as shown in Figure 10c. Brain-inspired neuromorphic chips were fabricated by nanocellulose-gated indium tin oxide as a synaptic transistor with an excellent, intelligent perception accuracy of 92.93%. At room temperature, the nanocellulose-gated indium tin oxide synaptic transistors could be dissolved in DI water, suggesting the possibility of green electronics in the future. In 2023, Wang et al. described a crystalline vertical organic electrochemical transistor (cv-OECTs) with the ability to sense, store memory, and process information that can be recorded in real-time cardiac disorder conditions through reservoir computing [97], as shown in Figure 10d. This synaptic transistor has a vertical transverse architecture with an amorphous and crystalline structure that can be used as a volatile receptor (for multi-model sensors) and non-volatile synapse (memory and processing), where 1-ethyl-3-methylimidazolium bis(trifluoromethylsulfonyl)imide ([EMIM^+^][TFSI^−^]): PVDF-HFP ion gel or aqueous solution are used as a gate dielectric vertical traverse OECT, developing a natural crossbar structure that can offer high amplifying in the volatile mode. Various synaptic performances and applications are summarized in Table 1.

Neurodegenerative disease-sensing is quite complicated compared to other perception disease diagnoses based on neuromorphic synaptic transistors. However, research is currently in process to develop an artificial synaptic transistor for application in neurodegenerative disease diagnosis.

**Table 1:** Summary of the device size, channel materials, and synaptic function for different types of synaptic transistors, including those used for neurodegenerative disease diagnosis.

Type of Transistor	Structural Feature	Active Layer	Dielectric Medium/EDL Formation	Synaptic Function	Biodegradable/ Biocompatible	Application	Refs.
FeTFT	Bottom gate	α-IGZO	Al:HfO2	STP, LTP	---	Potentiation/depression conditions	[1]
OECT	Bottom gate	DNTT	Dextran	EPSC, STP, LTP	Yes	Eco-friendly and bio-integrated organic electronics	[2]
ECFET	Bottom gate	ITO	Nanocellulose	EPSC, STP, LTP	Yes	Anxiety disorder	[3]
ECFET	Top electrode	MoS_2_	PVA hydrogel	EPSC, PPF	Yes	Cognitive systems	[11]
OECTs	Reference electrode	PEDOT:PSS	PBS	EPSC, IPSC, STP	---	Synaptic cooperation/neuroprosthetics	[12]
OECT	Top electrode	PEDOT:PSS	NaCl	EPSC	Yes	Synaptic cooperation	[23]
OECT	Bottom gate	ITO	PVA	EPSC, IPSC, STP	Yes	Biological interfaces	[24]
OECTs	Bottom gate	ITO	Chitosan	EPSC, LTP, STD, PPF	Yes	Multistore model brain memory	[46]
OECTs	Bottom gate	InZnO	Chitosan	EPSC, LTP, STD, PPF	Yes	Synaptic memory	[50]
ECFET	Bottom gate	P3HT	[EMI][TFSA]	EPSC, LTP, STD	----	Synaptic memory	[52]
EDLT	Reference electrode	MoS_2_	PVA	EPSC, LTP, STD	Yes	Electronic eyes	[84]
OECTs	Planar gate	PTBT	[EMIM+][TFSI−]):PVDF-HFP	EPSC, LTP, STD	Yes	ECG recording	[97]
OSCTs	Top electrode	SWCNTs	Chlorophyll-a	EPSC, LTP, LTM	Yes	Light-stimulated synaptic transistors	[100]
OSCTs	Top electrode	DNTT	Apple pectinPEM	EPSC, LTP, LTM	Yes	Biological interfaces	[101]

## 4. Conclusions and Future Perspectives

This review presents the organic hydrogel-gated field-effect transistors (HGFETs) that function through ion migration between an electrolyte and the channel. We outline five fundamental prerequisites for neuromorphic devices: synaptic plasticity, rapid switching capabilities, minimal energy usage, biodegradability, and biocompatibility with biological systems. The analyzed studies provided evidence that organic synaptic transistors exhibit a broad array of synaptic decay times with minimal energy consumption and can seamlessly interface with living organisms without causing any harm to cell structures in living cells. Furthermore, liquid electrolytes and hydrogel have gained extensive use in organic transistors due to their advantageous biotronic properties. As a result, biological interfacing is established without any interference. Nevertheless, to enhance device operational stability, particularly in the context of portable electronics, and achieve high-density integration, it becomes imperative to explore the incorporation of hydrogel electrolytes in organic synaptic transistors.

In future research, biodegradable/biocompatible bioinspired sensory systems should be developed to realize a more significant analysis of neurodegenerative disorders using ionotropic materials hydrogel as a gate dielectric of synaptic transistors with low-energy devices. For developing low energy consumption (E), artificial synaptic devices are to be focused on as follows:

- At the artificial synapse level, energy consumption (E) correlates directly with the applied voltage, drain current, and programming pulse duration. Artificial synapses leveraging ion migration with a high electric double-layer (EDL) capacitance in electrolyte gate insulators are favored to mitigate the applied voltage, as they necessitate only a low driving voltage. Additional strategies for minimizing energy consumption (E) involve reducing the duration of programming spikes and downsizing the dimensions of the devices.
- To address the issue of energy dissipation in sensing elements, a solution involves integrating artificial synapses with self-powered sensing components, directing the predominant energy dissipation solely to the synaptic devices. Alternatively, employing functioning artificial synapses endowed with sensing capabilities within a single device provides another avenue for mitigating energy consumption.
